# Reparación artroscópica de roturas del manguito rotador del hombro mediante técnica de una hilera frente a doble hilera. Revisión sistemática.

**DOI:** 10.31053/1853.0605.v80.n3.41161

**Published:** 2023-09-29

**Authors:** Ignacio Carbonel Bueno, Jorge Hernando Sacristán, Roberto García Pérez, Pablo Navarro López, Isabel Hernández Fernández, Jorge Ripalda Marín

**Affiliations:** 1 H U Miguel Servet de Zaragoza España; 2 HCU Lozano Blesa de Zaragoza. España; Universidad de Medicina de Zarargoza España

**Keywords:** lesiones del manguito de los rotadores, artroscopia, anclas para sutura, rotator cuff injuries, arthroscopy, suture anchors, lesões do manguito rotador, artroscopia, âncoras de sutura

## Abstract

**Objetivos:**

El propósito de este estudio es realizar una revisión sistemática de la literatura científica para comparar las técnicas de hilera simple y doble para los desgarros del manguito de los rotadores en términos de curación funcional, del dolor y estructural del tendón.

**Métodos:**

Para llevar a cabo la investigación se ha realizado una selección sistemática de artículos científicos consultando las bases de datos PubMed y The Cochrane Library. Los artículos incluidos en el presente estudio comparan las técnicas de una y dos hileras, con fecha de publicación entre 2016 y 2021.

**Resultados:**

Se observan diversos resultados en las escalas de valoración UCLA, SST, Constant y ASES y en el rango de movilidad, por lo que no se establece la superioridad de una técnica en cuanto a resultados funcionales. La técnica de doble hilera puede mostrar superioridad en la variable fuerza, aunque existe una variabilidad metodológica en su medición. La técnica de doble hilera puede estar relacionada con un dolor postoperatorio más intenso, mientras que la técnica de hilera simple con dolor residual en el hombro. Sin embargo, las variables fuerza y dolor muestran datos no homogéneos o coincidentes. Se establece con resultados estadísticamente significativos, siendo los estudios más homogéneos y coincidentes, la superioridad de la reparación en doble hilera en la cicatrización estructural del tendón y una menor tasa de re-desgarro que la técnica de hilera simple.

## Abstract (en)

**Purpose:**

The purpose of this study is to perform a systematic review of the scientific literature to compare single and double row techniques for rotator cuff tears in terms of functional, pain improvement and structural healing of the tendon.

**Methods:**

In order to carry out the research, a systematic selection of scientific articles has been made by consulting PubMed and The Cochrane Library databases. The articles included in the present study compare the single-row and double-row techniques, with publication date between 2016 and 2021.

**Results:**

Diverse results are observed in the UCLA, SST, Constant and ASES assessment scales and in the range of mobility, therefore, the superiority of one technique in terms of functional results is not established. The double row technique may show superiority in the strength variable, although there is a methodological variability in its measurement. The double-row technique may be related to more intense postoperative pain, while the single-row technique to residual shoulder pain. However, the variables strength and pain show non-homogeneous or coincident data. The superiority of the double row repair in the structural healing of the tendon and a lower re-tear rate than the single row technique is established with statistically significant results, being the most homogeneous and coincident studies.

## Resumo

**Objetivos:**

O objetivo deste estudo é realizar uma revisão sistemática da literatura científica para comparar as técnicas de fileira única e dupla para rupturas do manguito rotador em termos de cicatrização funcional, dolorosa e estrutural do tendão.

**Métodos:**

Para a realização da pesquisa, foi realizada uma seleção sistemática de artigos científicos por meio de consulta às bases de dados PubMed e The Cochrane Library. Os artigos incluídos no presente estudo comparam as técnicas de uma e duas linhas, com data de publicação entre 2016 e 2021.

**Resultados:**

São observados vários resultados nas escalas de avaliação UCLA, SST, Constant e ASES e na amplitude de movimento, não sendo estabelecida a superioridade de uma técnica em termos de resultados funcionais. A técnica de fileira dupla pode mostrar superioridade na variável força, embora haja variabilidade metodológica em sua mensuração. A técnica de fileira dupla pode estar associada a dor pós-operatória mais intensa, enquanto a técnica de fileira única a dor residual no ombro. No entanto, as variáveis força e dor apresentam dados não homogêneos ou coincidentes. Estabelece-se com resultados estatisticamente significativos, sendo os estudos mais homogêneos e coincidentes, a superioridade do reparo em dupla fileira na cicatrização estrutural do tendão e menor taxa de re-rasgo do que a técnica de fileira única.

**CONCEPTOS CLAVE**

Qué se sabe sobre el tema: La rotura del manguito de los rotadores tiene una gran importancia en la medicina actual debido a su alta frecuencia y al grado de discapacidad que produce. Su incidencia está en aumento debido, probablemente, al aumento de actividad por parte de la población y a la mayor disponibilidad de pruebas de imagen que permiten diagnosticar estas lesiones. El tratamiento de elección en las roturas de mayor grado es su reparación mediante cirugía artroscópica. El modelo de reparación clásico era la técnica de hilera simple pero posteriormente ha surgido la técnica de doble hilera, la cual incluye una fila más de anclajes con el fin de aumentar la estabilidad de la reparación.

Qué aporta este trabajo: A pesar de la existencia de numerosos estudios que comparan ambas técnicas artroscópicas, no parecen existir conclusiones acerca de la superioridad de una reparación frente a otra. Por ello, es necesario una revisión sistemática que reúna la mejor evidencia disponible y permita sacar conclusiones de la superioridad de una técnica sobre otra. El objetivo de esta revisión sistemática es responder a la pregunta: ¿Qué reparación artroscópica en las roturas del manguito de los rotadores obtiene mejores resultados en términos de funcionalidad, de disminución de dolor y de curación estructural validado mediante pruebas de imagen: la doble hilera o la hilera simple?

**Divulgación**

La patología del manguito de los rotadores del hombro es una de las más frecuentes del sistema musculoesquelético y es la causa más frecuente de dolor con origen en el hombro. El tratamiento de elección en las roturas de mayor grado es la reparación mediante técnicas diferentes de cirugía artroscópica: ‘’hilera simple’’ y ‘’doble hilera’’. El propósito de este estudio es realizar una revisión sistemática de la literatura científica para comparar ambas técnicas para los desgarros del manguito de los rotadores en términos de curación funcional, del dolor y estructural del tendón.

## Introducción

La patología del manguito de los rotadores es una de las más frecuentes del sistema musculoesquelético y es la causa más frecuente de dolor con origen en el hombro ^1 2^ .

El tratamiento de elección en las roturas de mayor grado es la reparación mediante cirugía artroscópica. El modelo de reparación clásico era la técnica de hilera simple pero posteriormente ha surgido la técnica de doble hilera. A pesar de la existencia de numerosos estudios que comparan ambas técnicas artroscópicas, no parecen existir conclusiones acerca de la superioridad de una frente a otra. Por ello, es necesario una revisión sistemática que reúna la mejor evidencia disponible y permita sacar conclusiones de la superioridad de una técnica sobre otra ^3 4^ .

La técnica de hilera simple consiste en una sola fila de anclajes dentro de la faceta lateral de inserción, de forma que, para realizar la reparación, las suturas se pasan a través del tendón ^5 6^ .

En la técnica de doble hilera una fila de anclajes se coloca en la faceta medial de la inserción, inmediatamente lateral a la superficie lateral de la cabeza humeral. Ambas suturas se pasan a través del tendón. Después, se coloca una fila lateral en la faceta lateral de la inserción, ligeramente proximal a la tuberosidad mayor. Las suturas de la fila lateral son usadas como suturas simples. Sólo una de las 2 suturas se pasa a través del tendón ^5 6^ .

## Objetivos

Nuestro trabajo pretende realizar una revisión sistemática de la bibliografía actual para ver si existe superioridad de una técnica frente a otra en términos funcionales, de mejoría del dolor y de curación estructural del tendón

## Material y métodos

### Búsqueda bibliográfica

La estrategia de búsqueda de la información se basó en el uso de las bases de datos PubMed y Cochrane Library.

En primer lugar, se realizó la búsqueda bibliográfica en Pubmed. Se utilizó el término MeSH: "Rotator Cuff Injuries", junto a términos libres como "Rotator cuff tear", "Rotator cuff repair", "Supraspinatus tendon tears", "Rotator cuff lesions", "Rotator cuff tendon repair", "Rotator cuff tears", "Single row", "Single", "Double row", "Suture bridge repair", "Transosseous equivalent", "Speed bridge technique", "Transosseous sutures", "Transosseous suture repair" y "Load-sharing rip-stop repair". Se incluyeron artículos presentados en los últimos 5 años en español o en inglés, obteniendo 32 resultados.

Posteriormente, la búsqueda de artículos de la literatura científica se realizó en la base de datos Cochrane Library. Se obtuvieron 11 resultados.

Para seleccionar entre los 43 artículos, se especifican a continuación los criterios de inclusión y exclusión de esta revisión sistemática.

### Criterios de inclusión

- Objetivo del estudio comparación de hilera simple frente a doble hilera desde el punto de vista funcional, del dolor o mediante pruebas de imagen,
- Idioma inglés o castellano.
- Tipos de estudios: metaanálisis, ensayos clínicos, ensayos clínicos aleatorizados, revisiones y estudios comparativos.
- Publicado en los últimos 5 años.
- No se establecieron restricciones en cuanto a la edad, el sexo, la etnia, comorbilidades ni tratamiento previos.

### Criterios de exclusión

- Tipos de estudios: comentarios, libros o editoriales.
- Estudios que no especifiquen las técnicas artroscópicas utilizadas o que no incluyan la técnica de simple o doble hilera.
- Estudios que comparen la hilera simple y la doble hilera desde un punto de vista económico.

En la revisión manual de los artículos, se descartaron 7 estudios por aparecer en ambas búsquedas. Se realizó una lectura del título y resumen y se descartaron 7 artículos en función de los criterios de inclusión y exclusión antes mencionados. El resultado fue la elección de 29 estudios.

**Figura 1:**
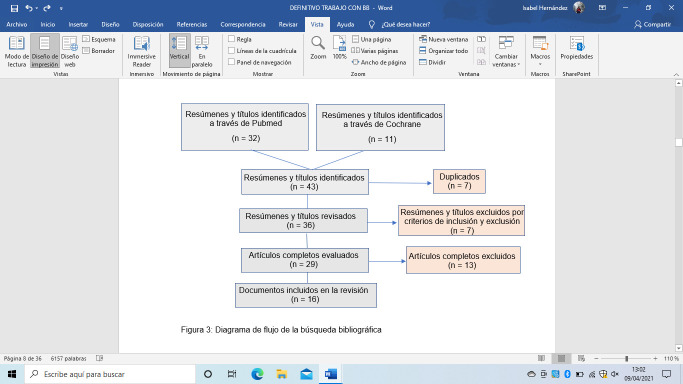
Diagrama de flujo sobre la búsqueda bibliográfica realizada

Todos los estudios se leyeron en su totalidad y al analizarlos más en profundidad se descartaron 13 artículos por no cumplir con los criterios del apartado anterior. Por tanto, se seleccionaron finalmente 16 estudios (Figura 1).

### Valoración crítica de la calidad de los artículos

La calidad de la evidencia científica de los artículos se clasificó siguiendo los criterios utilizados por la Agency for Healthcare Research and Quality (AHRQ) ^7^ , que proponen una serie de niveles de evidencia científica y grados de recomendación basados principalmente en el tipo de estudio evaluado, su diseño, metodología y aleatorización tabla 1.

**Tabla N° 1:** Distribución de las variables a estudio en los diferentes artículos incluidos en la revisión y nivel de evidencia en cada uno.

	ASES	SST	Constant	UCLA	Rango de movilidad	Fuerza	Dolor	Pruebas de imagen	Nivel de evidencia
Chen et al.	+	-	+	-	+	+	+	+	IIa(B)
Hantes et al.	-	-	+	+	-	-	-	+	IIa(B)
Hohmann et al.	-	-	-	-	-	-	-	+	III(B)
Imam et al.	-	-	+	+	+	-	+	-	Ib(A)
Jeong et al.	+	+	+	-	-	-	+	+	III(B)
Khoriati et al.	+	+	+	+	+	+	-	+	III(B)
Moon et al.	-	-	+	-	+	+	+	+	III(B)
Nicholas et al.	+	+	-	-	+	+	-	-	IIa(B)
Noyes et al.	+	+	-	-	+	-	+	+	III(B)
Panella et al.	-	-	+	+	+	+	+	-	III(B)
Plachel et al.	+	+	+	-	-	-	-	+	III(B)
Randelli et al.	-	-	+	-	+	-	+	+	Ib(A)
Sobhy et al.	+	-	+	+	-	-	-	+	Ia(A)
Spiegi et al.	+	+	+	+	+	+	+	+	III(B)
Tashjian et al.	+	+	-	-	+	+	+	+	III(B)
Yamakado et al.	-	-	-	+	+	-	+	+	Ib(A)

## Resultados

### ESTADÍSTICA DESCRIPTIVA GENERAL

#### Estudios

Se han analizado: 4 ensayos clínicos prospectivos, controlados y aleatorizados, 5 estudios de cohortes, 3 estudios comparativos retrospectivos, 2 metaanálisis y 2 revisiones sistemáticas.

Todos los estudios fueron realizados entre los años 2015 y 2020. En cuanto a su nivel de evidencia, se ha clasificado siguiendo los criterios AHRQ ^7^ , y su distribución es: 1 estudio de nivel Ia, 3 estudios de nivel Ib, 3 estudios de nivel IIa y 9 estudios de nivel III. Según el grado de recomendación, 4 estudios tienen una recomendación A y 12 estudios una recomendación B.

#### Tamaño muestral

El tamaño muestral varía entre un mínimo de 27 personas ^8^ y el máximo de 415 personas ^9^ . La media fue de 104 participantes, con un total de 1246. Estos datos no incluyen las revisiones sistemáticas y metaanálisis, donde el número de participantes es mucho mayor, con una media de 1149 participante.

#### Edad

Mínima de 49 años ^10^ y máxima de 66 años ^11^ , con una media de edad de 60 años por estudio. Al comparar la media de edad entre los grupos de hilera simple y doble hilera se encontró que no existían diferencias estadísticamente significativas en ningún estudio excepto en Nicholas et al ^12^ (p<0,05), donde los pacientes operados con la técnica de doble hilera eran mayores respecto al grupo de hilera simple. Randelli et al ^13^ fue el único estudio que no indicó la significación estadística.

#### Sexo

La distribución del sexo no mostró diferencias estadísticamente significativas en ningún estudio, por lo que la distribución de hombres y mujeres era homogénea. Los estudios de Tashjian et al ^14^ y de Randelli et al ^13^ no indicaron la significación estadística.

#### Tamaño de la lesión del manguito rotador

La diferenciación de los distintos tamaños de lesión no es igual, por lo que cada estudio hace sus propios grupos que no son directamente comparables. Además, este apartado no incluye significación estadística (tabla 5).

#### Periodo de seguimiento

Mínimo de 12 meses ^14^ y máximo de 12 años ^8^ , siendo la mediana de 2 años. La media es de 3,1 años, pero en este caso es menos representativa al ser sensible a los valores extremos (el segundo estudio con mayor periodo de seguimiento tuvo 3,5 años ^8^ .

Una vez analizada la estadística descriptiva general, se incluyen los apartados de análisis de losresultados funcionales, dolor y pruebas de imagen. Aunque los estudios incluidos en la presente revisión sistemática cumplen de forma estricta con los criterios de inclusión, no todos ellos incluyen todos los apartados que analizamos en este estudio. Se incluye en la tabla 6 los apartados incluidos en los diferentes estudios.

### ANÁLISIS DE LOS RESULTADOS FUNCIONALES

#### Análisis de las escalas de valoración

##### Medidas de los resultados

Para estudiar las diferencias funcionales entre la hilera simple y doble

hilera se han utilizado cuatro escalas de valoración validadas a nivel internacional: escala de ASES, SST, Constant y UCLA.

##### Resultados de los estudios

En la valoración de los resultados de la escala ASES ambas técnicas mostraban mejorías funcionales tras la reparación. Chen et al ^15^ , Jeong et al ^9^ , Sobhy et al ^16^ y Noyes et al ^11^ obtuvieron puntuaciones mayores con la técnica doble hilera. Sin embargo, los estudios de Nicholas et al ^12^ , Plachel et al ^8^ y Tashjian et al ^14^ , alcanzan mayores puntuaciones con la técnica de hilera simple. En todo caso, ningún estudio obtuvo diferencias estadísticamente significativas.

La revisión sistemática realizada por Spiegl et al ^17^ encontró que la técnica de doble hilera obtenía mejores puntuaciones en la escala ASES en las roturas mayores de 3 cm, siendo estas diferencias estadísticamente significativas.

En la valoración de los resultados de la escala SST se vio que la mayoría de los estudios obtuvieron puntuaciones similares en ambas técnicas con p>0,05. El único estudio que encontró diferencias estadísticamente significativas fue Tashjian et al ^14^ . La técnica hilera simple obtuvo una mejor puntuación entre el preoperatorio y el postoperatorio (p=0,03).

En la valoración de la escala Constant ningún estudio encontró diferencias estadísticamente significativas.

Por último, en la valoración de la escala UCLA, los estudios realizados por Khoriati et al ^18^ , Sobhy et al ^16^ y Spiegl et al ^17^ encontraron diferencias estadísticamente significativas entre ambas técnicas artroscópicas. En primer lugar, la revisión sistemática de Khoriati et al ^18^ halló mejores puntuaciones en la escala UCLA con la técnica doble hilera en todos los tamaños de rotura del manguito de los rotadores. El metaanálisis de Sobhy et al ^16^ encontró mejores puntuaciones con la técnica artroscópica de doble hilera (p=0,004). Por último, la revisión sistemática realizada por Spiegl et al ^17^ mostró que la técnica de doble hilera obtenía mejores puntuaciones en la escala UCLA en las roturas del manguito mayores de 3 cm.

En la tabla 2 se recogen las puntuaciones que cada grupo obtuvo en las diferentes escalas y el valor p de significación estadística.

**Tabla 2:** Puntuaciones de los grupos de la técnica hilera simple y de la técnica doble hilera en las diferentes escalas de valoración.

	ASES			SST			Constant			UCLA		
	Hilera simple	Doble hilera	p	Hilera simple	Doble hilera	p	Hilera simple	Doble hilera	p	Hilera simple	Doble hilera	p
Chen et al.	88,3 ∓ 9,7	90,1 ∓ 11,3	n.s	-	-		75.4 ∓ 6.6	76.1 ∓ 6.9	n.s	-	-	
Hantes et al.	-	-		-	-		86,3	90,3	0.25	30,1	32,2	0.134
Imam et al.	-	-		-	-		79.4 ∓ 8.3	77.6 ∓ 9.4	0.70	31 ∓ 2,3	31.3 ∓ 2,2	0.409
Jeong et al.	79,91 ∓ 17,52	81,41 ∓ 18,11	0.21	9 ∓ 2,97	9,35 ∓ 2,75	0.55	76.39 ∓ 15.88	75 ∓ 16.09	0.23	-	-	
Khoriati et al.	No diferencias			No diferencias			No diferencias			Doble hilera mejor en > 3cm		
Moon et al.	-	-		-	-		80 ∓ 6,8	79.8 ∓ 4.4	0.792	-	-	
Nicholas et al.	92 ∓ 12	87 ∓ 12	0.21	11,3 ∓ 1	11,4 ∓ 1	0.76	-	-		-	-	
Noyes et al.	78,1	89,5	0.16	8,6	10,3	0.38	-	-		-	-	
Panella et al.	-	-		-	-		81,5 ∓ 7,8	86,1 ∓ 4,5	0.36	28.5 ∓ 2.4	30.4 ∓ 1.5	0.21
Plachel et al.	90 ∓ 21	83 ∓ 24	0.34	10 ∓ 3	10 ∓ 3	0.25	85 ∓ 19	80 ∓ 20	0.45	-	-	
Randelli et al.	-	-		-	-		72.3	69,9	0.25	-	-	
Sobhy et al.	Doble hilera ligeramente mejor		0.10	-	-		Doble hilera ligeramente mejor		0.32	Doble hilera mejor puntuación		0.004
Spiegl et al.	Doble hilera mejor en > 3cm			No diferencias			No diferencias			Doble hilera mejor en > 3cm		
Tashjian et al.	96	87,3	0.73	11,7	10,5	0.03	-	-		-	-	
Yamakado et al.	-	-		-	-		-	-		33 ∓ 3.1	33.4 ∓ 3.3	0.58

#### Análisis del rango de movilidad

##### Medidas de resultados

Se analizaron los grados de movilidad del hombro para la flexión, abducción, rotación interna y rotación externa. Para su medición se utilizó un goniómetro estándar.

Los estudios realizados por Chen et al ^15^ , Moon et al ^19^ , Nicholas et al ^12^ , Tashjian et al ^14^ y Yamakado et al ^20^ expresan los resultados en grados. Por otra parte, el estudio de Panella et al ^21^ expresa el resultado dentro de la puntuación de la escala Constant, valorándolo con una puntuación máxima total de 40. De esta forma, desconocemos la puntuación individual de cada plano del movimiento y el resultado es un total de puntos tras la valoración de los movimientos. Por último, El estudio de Noyes et al ^11^ valoró la flexión y la rotación externa mediante grados, sin embargo, la rotación interna la valoró a través de nivel espinal más cercano.

##### Resultados de los estudios

Imam et al ^5^ encontró que la técnica artroscópica doble hilera conseguía un mayor rango de movilidad frente a la técnica hilera simple tanto en el movimiento de flexión (p=0,001) como en el movimiento de rotación externa (p=0,010). Sin embargo, no se encontraron diferencias entre ambas técnicas en la rotación interna (p=0,14) y en la abducción (p=0,12).

Nicholas et al ^12^ encontró que los pacientes operados con la técnica artroscópica de doble hilera tuvieron una pérdida de 7 grados en el movimiento de rotación externa frente a su movilidad en el preoperatorio (p<0,05). Durante el seguimiento, los pacientes con reparaciones de doble hilera tenían 10 grados menos en rotación externa respecto a los pacientes con reparaciones de hilera simple (p<0,01).

Los restantes estudios no encontraron diferencias estadísticamente significativas en el rango de movilidad entre las dos técnicas (Tabla 3).

**Tabla 3:** Rangos de movilidad en flexión, abducción, rotación interna y externa de los grupos de hilera simple y doble hilera.

	Flexión			Abducción			Rotación interna			Rotación externa		
	Hilera simple	Doble hilera	p	Hilera simple	Doble hilera	p	Hilera simple	Doble hilera	p	Hilera simple	Doble hilera	p
Chen et al.	161º ∓ 10º	162º ∓ 9º	n.s	158º ∓ 15º	160º ∓ 12º	n.s	8º ∓ 3º	9º ∓ 3º	n.s	59º ∓ 12º	56º ∓ 14º	n.s
Imam et al.	Mejor doble hilera		0.001	No diferencias		0.12	No diferencias		0.014	Mejor doble hilera		0.010
Khoriati et al.	No diferencias			No diferencias			No diferencias			No diferencias		
Moon et al.	153º ∓ 15º	154º ∓ 14º	0.870	117º ∓ 19º	118º ∓ 23º	0.723	50º ∓ 11º	50º ∓ 10º	1	73º ∓ 15º	74º ∓ 14º	0.663
Nicholas et al.	170º ∓ 3º	162º ∓ 3º	0.09				57º ∓ 15º	50º ∓ 11º	0.17	67º ∓ 10º	59º ∓ 10º	0.02
Noyes et al.	144o	153o	0.776				L3	L1	0.178	48o	39o	0.784
Panella et al.	34,8/40	37,2/40	0.2	34,8/40	37,2/40	0.2	34,8/40	37,2/40	0.2	34,8/40	37,2/40	0.2
Spiegl et al.	No diferencias			No diferencias			No diferencias			No diferencias		
Tashjian et al.	168º ∓ 5º	160º ∓ 29º	0.22									
Yamakado et al.	161o	159o	0.34							49o	52o	0.37

#### Análisis de la fuerza

##### Medidas de resultados

Nicholas et al ^12^ calculó la fuerza en abducción y rotación externa utilizando los test de lata vacía y lata llena. La prueba de lata vacía se pide que ponga el brazo 90 º de abducción, flexión anterior de 30º y rotación interna. La prueba de lata llena se pide que ponga el brazo en 90° de abducción y 45° de rotación externa.

Tashjian et al ^14^ midió la fuerza de elevación hacia adelante a 90⁰ de elevación en el plano escapular.

Panella et al ^21^ utilizó como medición la fuerza del supraespinoso (brazo a 90° de abducción, codo completamente extendido, mano en pronación).

Moon et al ^19^ midió la fuerza muscular cuantitativamente con un dinamómetro en flexión, rotación interna y rotación externa.

Chen et al ^15^ utilizó como medida la fuerza de abducción. Se evaluó en ambos lados con el paciente sentado con el brazo en abducción de 90°. La medida que se utilizó para comparar las técnicas de hilera simple y doble hilera fue la diferencia de fuerza entre el brazo con la lesión y el contralateral.

##### Resultados de los estudios

En el estudio de Nicholas et al ^12^ la fuerza mejoró en las 4 pruebas desde el preoperatorio hasta el seguimiento final. Sin embargo, los resultados no mostraron diferencias estadísticamente significativas entre grupos (p=0.23-0.75). No obstante, en ambos grupos la fuerza mejoró en un 54% en la prueba lata vacía, 66% en la prueba lata llena, 47% en abducción y 54% en rotación externa.

Panella et al ^21^ encontró que la doble hilera obtenía mejores resultados en términos de fuerza respecto a la hilera simple (p=0,01).

Tashjian et al ^14^ ,Moon et al ^19^ , Chen et al ^15^ , Khoriati et al ^18^ y Spiegl et al ^17^ no encontraron diferencias estadísticamente significativas en la fuerza entre la hilera simple y doble hilera.

#### Análisis del dolor

##### Medidas de resultados

Las dos escalas que se han utilizado en los estudios para evaluar el dolor han sido la escala analógica visual (EVA) y la escala numérica (EN).

La escala analógica visual ha sido utilizada por los estudios de Imam et al ^5^ , Noyes et al ^11^ , Tashjian et al ^14^ , Jeong et al ^9^ , Moon et al ^19^ , Chen et al ^15^ y Yamakado et al ^20^ .

La escala numérica ha sido utilizada por Randelli et al ^13^ .

##### Resultados de los estudios

Imam et al ^5^ encontró una diferencia a las 24 horas de seguimiento en la puntuación de la EVA para los desgarros < 3 cm y > 3 cm a favor de hilera simple. Estas diferencias fueron estadísticamente significativas (p=0,019) y (p<0,01) respectivamente. A las 6 semanas, no se encontraron estas diferencias.

Chen et al ^15^ observó que una proporción significativamente mayor del grupo de hilera simple mostró dolor de hombro residual en comparación con el grupo de doble hilera (p=0,022). También se vio que hubo dolor más intenso después de la cirugía en el grupo de doble hilera respecto a hilera simple tras 1 semana (p<0,001) y a los 3 meses (p=0,041), mientras que la intensidad del dolor fue similar a los 6, 12 y 24 meses de seguimiento.

Los estudios restantes no encontraron diferencias estadísticamente significativas entre las dos técnicas (tabla 5).

**Tabla 4:** Resultados de la variable dolor. Se incluyen las mediciones en los grupos de hilera simple y doble hilera en el preoperatorio y postoperatorio

			Doble hilera		
	Hilera simple				
	Preoperatorio	Postoperatorio	Preoperatorio	Postoperatorio	p
Chen et al.	5,5/10	2,61/10	5,6/10	3,21/10	0.04
Imam et al.	53,88/100	13,4/100	56,45/100	12,2/100	0.01
Jeong et al.	5,75/10	1,94/10	5,59/10	1,86/10	0.34
Moon et al.	4,1/10	0,3/10	4,5/10	0,4/10	0.49
Noyes et al.	5,6/10	1,9/10	4,8/10	0,8/10	0.83
Panella et al.		11,3/15		10,3/15	0.38
Randelli et al.	5,1/10	1,3/10	5,7/10	1,2/10	0.91
Spiegl et al.	No diferencias		No diferencias		
Tashjian et al.	3,3/10	0,08/10	4,6/10	0,9/10	0.47
Yamakado et al.	62/100	7/100	63/15	6/100	0.38

**Tabla 5:** Resultados de prueba de imagen.

	Tasa de curación			Clasificación Sugaya			Tasa de re-desgarro			Patrones de re-desgarro		
				(I/II/III/IV/V)						(Tipo 1 / tipo 2)		
	Hilera simple	Doble hilera	p	Hilera simple	Doble hilera	p	Hilera simple	Doble hilera	p	Hilera simple	Doble hilera	p
Chen et al.	89 %	94 %		9/8/14/11/0	15/24/8/6/0	0.028	11 %	6 %	n.s			
Hantes et al.	61 %	84 %	0.031	2/10/9/9/4	7/12/8/3/2		39 %	16 %	0.027			
Hohmann et al.	Mayor en doble hilera						Mayor en hilera simple			No diferencias estadísticamente significativas		
Jeong et al.	91 %	95.1%					8.94%	4,88 %	0.105	14-mar	04-jul	0.013
Khoriati et al.	Mayor en doble hilera						Mayor en hilera simple					
Moon et al.	70.2%	87.6%					29.8%	12.4%	0.002			
Noyes et al.	11 %	53 %	0.010				89 %	47 %				
Plachel et al.	67 %	45 %		0/25/50/13/12	0/27/4/27/0	0.41	33 %	55 %	0.37			
Randelli et al.	88 %	87 %	0.81	7/17/7/3/1	5/17/4/4/0	0.81	12 %	13 %				
Sobhy et al	68 %	81 %					32 %	19 %	0.001			
Spiegl et al.	Mayor en doble hilera						Mayor en hilera simple					
Tashjian et al.	78 %	71 %	0.7	9/3/2/1/3	6/6/3/2/4		22 %	29 %		01-feb	02-mar	0.99
Yamakado et al.	97.9%	93.5%		25/15/5/1/0	30/11/2/3/0	0.35	2.1%	6.5%	0.31	0/1	01-feb	

#### Análisis de pruebas de imagen

##### Medidas de resultados

Las pruebas de imagen utilizadas para evaluar la estructura del tendón variaron en los estudios. Por un lado, Chen et al ^15^ , Hantes et al ^10^ , Jeong et al ^9^ , Khoriati et al ^18^ , Randelli et al ^13^ , Tashjian et al ^14^ y Yamakado et al ^20^ utilizaron la Resonancia Magnética (RM). Por otro lado, Noyes et al ^11^ y Plachel et al ^8^ utilizaron la ecografía. Moon et al ^19^ utilizó la Tomografía Computarizada (TC) y la ecografía. Sobhy et al ^16^ utilizó la RM y la ecografía.

Se analizaron cuatro parámetros diferentes: la tasa de curación del tendón, el tipo de curación según la clasificación de Sugaya ^22^ , la tasa de re-desgarro y los patrones de re-desgarro según Cho et al ^23^ .

##### Resultados de los estudios

Chen et al ^15^ encontró un total de 17 nuevos desgarros (16,2%). La diferencia en las tasas de desgarro de los grupos no fue estadísticamente significativa, mientras que la integridad del tendón evaluada utilizando la clasificación de Sugaya fue significativamente diferente entre los grupos (p=0,028). Este estudio demostró una integridad del tendón significativamente mejor en el grupo de doble hilera y concluyó que la técnica doble hilera obtiene una mejor curación a corto plazo después de la cirugía.

La RM posoperatoria en el estudio de Hantes et al ^10^ reveló una tasa de curación del tendón significativamente mayor después de la reparación de doble hilera comparado con la reparación de una sola hilera (p=0,031) y una tasa de re-desgarro mayor con la técnica de hilera simple (p=0,027).

## Discusión

En la actualidad, las roturas del manguito rotador generan un gran interés. La bibliografía actual que compara las técnicas de hilera simple y doble hilera es escasa. Por ello, el objetivo de esta revisión es reunir la mejor evidencia posible comparando ambas técnicas.

Las escalas de valoración funcionales incluidas en los artículos fueron ASES, SST, Constant y UCLA. La mayoría de los artículos no encontraron diferencias significativas. Sin embargo, las revisiones sistemáticas y los metaanálisis pueden aportar algún dato interesante. ASES y UCLA muestran superioridad en la técnica doble hilera ^16-18^ , la escala SST en la hilera simple ^14^ .

El rango de movilidad fue valorado en flexión, abducción, rotación interna y rotación externa. La mayoría de artículos no incluían resultados en los cuatro planos ni encontraron diferencias estadísticamente significativas. Los estudios de Imam et al ^5^ y Nicholas et al ^12^ encontraron diferencias entre ambas técnicas, el primero a favor de la doble hilera en flexión y rotación externa y el segundo a favor de la hilera simple en rotación externa. Por lo tanto, no es posible establecer la superioridad en términos de balance articular.

La fuerza fue evaluada por una gran variedad de métodos. En la literatura revisada no existe consenso de cuál es el mejor método de evaluación. En la mayoría de artículos no se encontraron diferencias significativas entre ambos grupos. Panella et al ^21^ fue el único estudio que encontró una mayor fuerza en el grupo doble hilera, con diferencias estadísticamente significativas.

El dolor fue evaluado en la mayoría mediante la escala EVA. Aunque la gran mayoría de estudios no encontraron diferencias estadísticamente significativas. Imam et al ^5^ encontró que la doble hilera se relaciona con un dolor postoperatorio superior. Chen et al ^15^ encontró que la técnica doble hilera se relaciona con una intensidad de dolor mayor en los tres primeros meses tras cirugía. Sin embargo, también observó que la técnica hilera simple se relacionaba con dolor de hombro residual tras 2 años de la operación en comparación con la técnica doble hilera.

Los resultados en las pruebas de imagen es el punto donde se muestra mayor coincidencia entre los estudios incluidos. Parece que la técnica doble hilera demuestra una mayor curación del tendón y una menor tasa de re-desgarro.

Jeong et al ^9^ mostró una mejor curación del tendón en desgarros > 3cm con la reparación doble hilera por lo que sugiere que en las lesiones de este tamaño es conveniente la utilización de la doble hilera. Igualmente, Moon et al ^19^ mostró una mayor tasa de re-desgarro en hilera simple en comparación con la técnica de doble hilera en las lesiones de medio y gran tamaño. Es probable que el mayor número de suturas en el área de la huella utilizadas en la doble hilera contribuyan a mejorar la continuidad posoperatoria del tendón en comparación con la técnica de una sola hilera.

Noyes et al ^11^ y Hantes et al ^10^ observaron que la tasa de curación del tendón fue mayor en el grupo de reparación de doble hilera. Chen et al ^15^ demostró una integridad del tendón significativamente mayor en el grupo de doble hilera. Chen et al ^15^ también mostró una relación entre la técnica de hilera simple y un mayor dolor de hombro residual. Este estudio sugiere que la menor calidad del tendón y la mayor retracción del mismo son factores de riesgo para el dolor de hombro residual.

La gran mayoría de artículos revisados muestran mejores resultados con la técnica doble hilera en la curación estructural del tendón. Sin embargo, no se puede establecer la superioridad de una técnica frente a otra en las puntuaciones de las escalas o en el rango de movilidad. Únicamente en un estudio se observa la superioridad de la técnica de doble hilera en la variable fuerza en comparación con la hilera simple. Por tanto, estos resultados muestran que la curación estructural del tendón no se relaciona directamente con mejorías funcionales. El metaanálisis de Sobhy et al ^16^ muestra una relación clara entre la superioridad funcional y la óptima integridad del tendón con resultados superiores en la técnica doble hilera.

Se puede decir que se ha cumplido el objetivo de este estudio y la hipótesis que se había propuesto ha sido confirmada. Sin embargo, son necesarios ensayos y metaanálisis futuros más potentes y de alto nivel de evidencia que evalúen si la establecida superioridad de la técnica doble hilera en términos de curación estructural del tendón se relaciona con mejorías funcionales.

## Limitaciones

La primera limitación fue la escasez de ensayos clínicos aleatorizados o estudios con nivel de evidencia alta. Es necesario realizar estudios de mayor calidad para poder establecer la mayor evidencia posible.

En segundo lugar, no existen unas medidas estándar normalizadas utilizadas en los diferentes estudios a la hora de evaluar las diferentes variables (fuerza muscular, rango de movilidad). De esta forma, es difícil la comparación entre los artículos y el extraer conclusiones.

Finalmente, tampoco se pudo estratificar los resultados según el tamaño inicial de la lesión, debido a la escasez de estudios que comparan la hilera simple y la doble hilera según el tamaño del desgarro.

## Conclusión

- Aunque artículos incluidos en el presente estudio afirman que la técnica doble hilera obtiene mejores puntuaciones en las escalas ASES y UCLA y la técnica hilera simple en la escala SST, estos resultados no son coincidentes ni homogéneos. Bajo nuestro punto de vista ninguna técnica es superior en cuanto a la funcionalidad valorada mediante escalas de valoración.
- En la comparativa de los resultados de rango de movilidad no se puede expresar superioridad de una técnica sobre otra.
- La comparación en términos de fuerza no establece la superioridad de ninguna técnica. A pesar de encontrar una mejoría en la técnica doble hilera, este resultado es aislado y serían necesarios estudios de mayor calidad.
- La técnica doble hilera se puede relacionar con un dolor posoperatorio precoz más intenso y la hilera simple con el dolor de hombro residual. Dado que esta afirmación se ha extraído de dos artículos, consideramos necesaria la realización de ensayos clínicos aleatorizados con un seguimiento largo antes de confirmar esta premisa.
- La reparación artroscópica doble hilera obtiene una mayor tasa de curación estructural en las pruebas de imagen en comparación con la reparación de hilera simple.

En definitiva, creemos que es necesaria la realización de estudios de mayor calidad para confirmar estas afirmaciones.

## References

[1] Iriarte Posse I, Pedret Carballido C, Balius Matas R, Cerezal Pesquera L (2020). Ecografía Musculoesquelética: Exploración anatómica y Patología.

[2] Osma Rueda JL, Carreño Mesa FA (2016). Manguito de los rotadores: epidemiología, factores de riesgo, historia natural de la enfermedad y pronóstico. Revisión de conceptos actuales. Revista Colombiana de Ortopedia y Traumatología.

[3] Wieser K, Bouaicha S, Grubhofer F (2019). Rotatorenmanschettenruptur: Wann ist die konservative und wann die operative Therapie indiziert?. Praxis (Bern 1994).

[4] Dang A, Davies M (2018). Rotator Cuff Disease: Treatment Options and Considerations. Sports Med Arthrosc Rev.

[5] Imam M, Sallam A, Ernstbrunner L, Boyce G, Bardakos N, Abdelkafy A, Moussa M, Ghazal MA (2020). Three-year functional outcome of transosseous-equivalent double-row vs. single-row repair of small and large rotator cuff tears: a double-blinded randomized controlled trial. J Shoulder Elbow Surg.

[6] Arroyo-Hernández M, Mellado-Romero MA, Páramo-Díaz P, Martín-López CM, Cano-Egea JM, Vila-Rico J (2015). Estudio comparativo de la reparación de la rotura de espesor completo del supraespinoso mediante técnica "hilera simple" o "suture bridge". Acta Ortop Mex. Acta Ortop Mex.

[7] Marzo-Castillejo M, Alonso-Coello P, Rotaeche del Campo R (2006). Cómo clasificar la calidad de la evidencia y la fuerza de las recomendaciones?. Aten Primaria.

[8] Plachel F, Siegert P, Rüttershoff K, Thiele K, Akgün D, Moroder P, Scheibel M, Gerhardt C (2020). Long-term Results of Arthroscopic Rotator Cuff Repair: A Follow-up Study Comparing Single-Row Versus Double-Row Fixation Techniques. Am J Sports Med.

[9] Jeong JY, Park KM, Sundar S, Yoo JC (2018). Clinical and radiologic outcome of arthroscopic rotator cuff repair: single-row versus transosseous equivalent repair. J Shoulder Elbow Surg.

[10] Hantes ME, Ono Y, Raoulis VA, Doxariotis N, Venouziou A, Zibis A, Vlychou M (2018). Arthroscopic Single-Row Versus Double-Row Suture Bridge Technique for Rotator Cuff Tears in Patients Younger Than 55 Years: A Prospective Comparative Study. Am J Sports Med.

[11] Noyes MP, Ladermann A, Denard PJ (2017). Functional outcome and healing with a load- sharing rip-stop repair compared with a single-row repair for large and massive rotator cuff tears. Arthroscopy.

[12] Randelli P, Stoppani CA, Zaolino C, Menon A, Randelli F, Cabitza P (2017). Advantages of Arthroscopic Rotator Cuff Repair With a Transosseous Suture Technique: A Prospective Randomized Controlled Trial. Am J Sports Med.

[13] Tashjian RZ, Granger EK, Chalmers PN (2018). Healing Rates and Functional Outcomes After Triple-Loaded Single-Row Versus Transosseous-Equivalent Double-Row Rotator Cuff Tendon Repair. Orthop J Sports Med.

[14] Chen Y, Li H, Qiao Y, Ge Y, Li Y, Hua Y, Chen J, Chen S (2019). Double-row rotator cuff repairs lead to more intensive pain during the early postoperative period but have a lower risk of residual pain than single-row repairs. Knee Surg Sports Traumatol Arthrosc.

[15] Sobhy MH, Khater AH, Hassan MR, El Shazly O (2018). Do functional outcomes and cuff integrity correlate after single- versus double-row rotator cuff repair? A systematic review and meta-analysis study. Eur J Orthop Surg Traumatol.

[16] Spiegl UJ, Euler SA, Millett PJ, Hepp P (2016). Summary of Meta-Analyses Dealing with Single-Row versus Double-Row Repair Techniques for Rotator Cuff Tears. Open Orthop J.

[17] Khoriati AA, Antonios T, Gulihar A, Singh B (2019). Single Vs Double row repair in rotator cuff tears - A review and analysis of current evidence. J Clin Orthop Trauma.

[18] Moon N, Lee SJ, Kwak SH, Lee E, Jeong HS, Suh KT (2018). Clinical and radiologic outcomes after arthroscopic rotator cuff repair: Single-row versus Speed-Bridge technique. Acta Orthop Belg.

[19] Yamakado K (2019). A Prospective Randomized Trial Comparing Suture Bridge and Medially Based Single-Row Rotator Cuff Repair in Medium-Sized Supraspinatus Tears. Arthroscopy.

[20] Panella A, Amati C, Moretti L, Damato P, Notarnicola A, Moretti B (2016). Single-row and transosseous sutures for supraspinatus tendon tears: a retrospective comparative clinical and strength outcome at 2-year follow-up. Arch Orthop Trauma Surg.

[21] Vicente-Herrero MT, Delgado-Bueno S, Bandrés-Moyá F, Ramírez-Iñiguez-de-la-Torre MV, Capdevilla-García L (2018). Valoración del dolor. Revisión comparativa de escalas y cuestionarios. Rev. Soc. Esp. Dolor.

[22] Sugaya H, Maeda K, Matsuki K, Moriishi J (2005). Functional and structural outcome after arthroscopic full-thickness rotator cuff repair: single-row versus dual-row fixation. Arthroscopy.

[23] Hohmann E, König A, Kat CJ, Glatt V, Tetsworth K, Keough N (2018). Single- versus double-row repair for full-thickness rotator cuff tears using suture anchors. A systematic review and meta-analysis of basic biomechanical studies. Eur J Orthop Surg Traumatol.

